# Implementation of family psychosocial risk assessment in pediatric cancer with the Psychosocial Assessment Tool (PAT): study protocol for a cluster-randomized comparative effectiveness trial

**DOI:** 10.1186/s13012-020-01023-w

**Published:** 2020-07-29

**Authors:** Anne E. Kazak, Janet A. Deatrick, Michele A. Scialla, Eric Sandler, Rebecca E. Madden, Lamia P. Barakat

**Affiliations:** 1 Nemours Children’s Health System, Wilmington, DE USA; 2 Sidney Kimmel Medical School of Thomas Jefferson University, Philadelphia, PA USA; 3 University of Pennsylvania School of Nursing, Philadelphia, PA USA; 4 A.I. du Pont Hospital for Children, Wilmington, DE USA; 5 Nemours Children’s Clinic, Jacksonville, FL USA; 6 The Children’s Hospital of Philadelphia, Philadelphia, PA USA; 7 Perelman School of Medicine of the University of Pennsylvania, Philadelphia, PA USA

**Keywords:** Pediatric cancer, Families, Psychosocial, Risk, Screening, Implementation

## Abstract

**Background:**

Childhood cancer affects and is affected by multiple levels of the social ecology, including social and relational determinants of health (e.g., economic stability, housing, childcare, healthcare access, child and family problems). The 2015 Standards of Psychosocial Care in Pediatric Cancer outline optimal psychosocial care sensitive to these ecological factors, starting with assessment of psychosocial healthcare needs to promote medical and psychosocial outcomes across all children with cancer. To address the first standard of family psychosocial assessment, the Psychosocial Assessment Tool (PAT) is a validated screener ready for broad implementation.

**Method:**

The PAT will be implemented across a national sample of 18 pediatric cancer programs ranging in size (annual new patients) in a mixed methods, comparative effectiveness study, guided by the Interactive Systems Framework for Dissemination and Implementation, comparing two implementation strategies. It is hypothesized that implementation will be more successful at the patient/family, provider, and institutional level when training (strategy I) is combined with implementation expanded resources (strategy II). There are three aims: (1) Refine the two implementation strategies using semi-structured qualitative interviews with 19 stakeholders including parent advocates, providers, pediatric oncology organization representatives, healthcare industry leaders; (2) Compare the two theoretically based and empirically informed strategies to implement the PAT in English and Spanish using a cluster-randomized controlled trial across 18 sites. Stratified by size, sites will be randomized to cohort (3) and strategy (2). Outcomes include adoption and penetration of screening (patient/family), staff job satisfaction/burnout (provider), and cost-effective use of resources consistent with family risk (institution); (3) Based on the results of the trial and feedback from the first and second aim, we will develop and disseminate a web-based PAT Implementation Toolkit.

**Discussion:**

Use of the PAT across children’s cancer programs nationally can achieve the assessment standard and inform equitable delivery of psychosocial care matched to family need for all patients.

**Trial Registration:**

ClinicalTrials.gov, NCT04446728, registered 23 June 2020

Contributions to the literature

- First study to apply dissemination and implementation methods to psychosocial screening in pediatric populations, specifically children with cancer and their families
- Application of rigorous implementation methods to pediatric health care setting
- Test of implementation of psychosocial screening to reduce health disparities

## Background

The diagnosis and treatment of pediatric cancer affects and is affected by multiple levels of the social ecology, including patient and caregiver physical and psychosocial health. Particularly at risk are families with limited instrumental (i.e., financial) and social resources and pre-existing child and family problems. Institute of Medicine Reports [1, 2] and the Standards of Psychosocial Care in Pediatric Cancer [3] call for improvement in delivery of psychosocial care. The standards outline evidence-based care for *all* patients and families to improve health, increase access to care, and reduce health disparities by decreasing distress, addressing risks, and improving quality of life. The first standard is “youth with cancer and their family members should routinely receive systematic assessment of their psychosocial healthcare needs” [4]. Universal screening at diagnosis fosters early identification of psychosocial risks and provides the opportunity to match psychosocial care to the level of family need for more equitable, effective, and integrated services. Confirming this, in qualitative interviews with multidisciplinary healthcare providers regarding the implementation of screening, the overarching theme was that screening all families is important because it facilitates clinical care and partnerships that can improve outcomes especially for those at risk for disparities. However, few programs offer such care in an efficient, comprehensive, consistent manner [5, 6], highlighting critical gaps in care that can magnify health disparities.

This study addresses this critical gap in the delivery of care to our diverse population of children with cancer and their families by evaluating two approaches to implementing an evidence-based, parent report screener of family psychosocial risk in English and Spanish—the Psychosocial Assessment Tool (PAT) [7, 8]. Risk screening initiates a process of preventive interventions across cancer treatment and facilitates access to evidence-based psychosocial care for children, potentially preventing increased distress and long-term limitations to health-related quality of life [9, 10]. Universal, systematic screening assures that assessments are integrated and resources meet the needs of *all* children with cancer and their families. However, barriers to universal, systematic screening and linked evidence-based care have been identified. The Preparing to Implement the Psychosocial Standards–Current Staffing and Services (PIPS-CSS) study of 144 US pediatric cancer programs conducted to prepare for broad implementation of the standards [5, 11] found that there are challenges and inconsistent interpretations of psychosocial care [5, 11]. Similarly, data from the Children’s Oncology Group (COG) [6] and a national survey of social workers [12] demonstrated inconsistent and often inadequate services. Barriers to implementation are evident, and providers note the importance of training in terms of how screening is accomplished [13].

### The Psychosocial Assessment Tool (PAT)

**Fig. 1:**
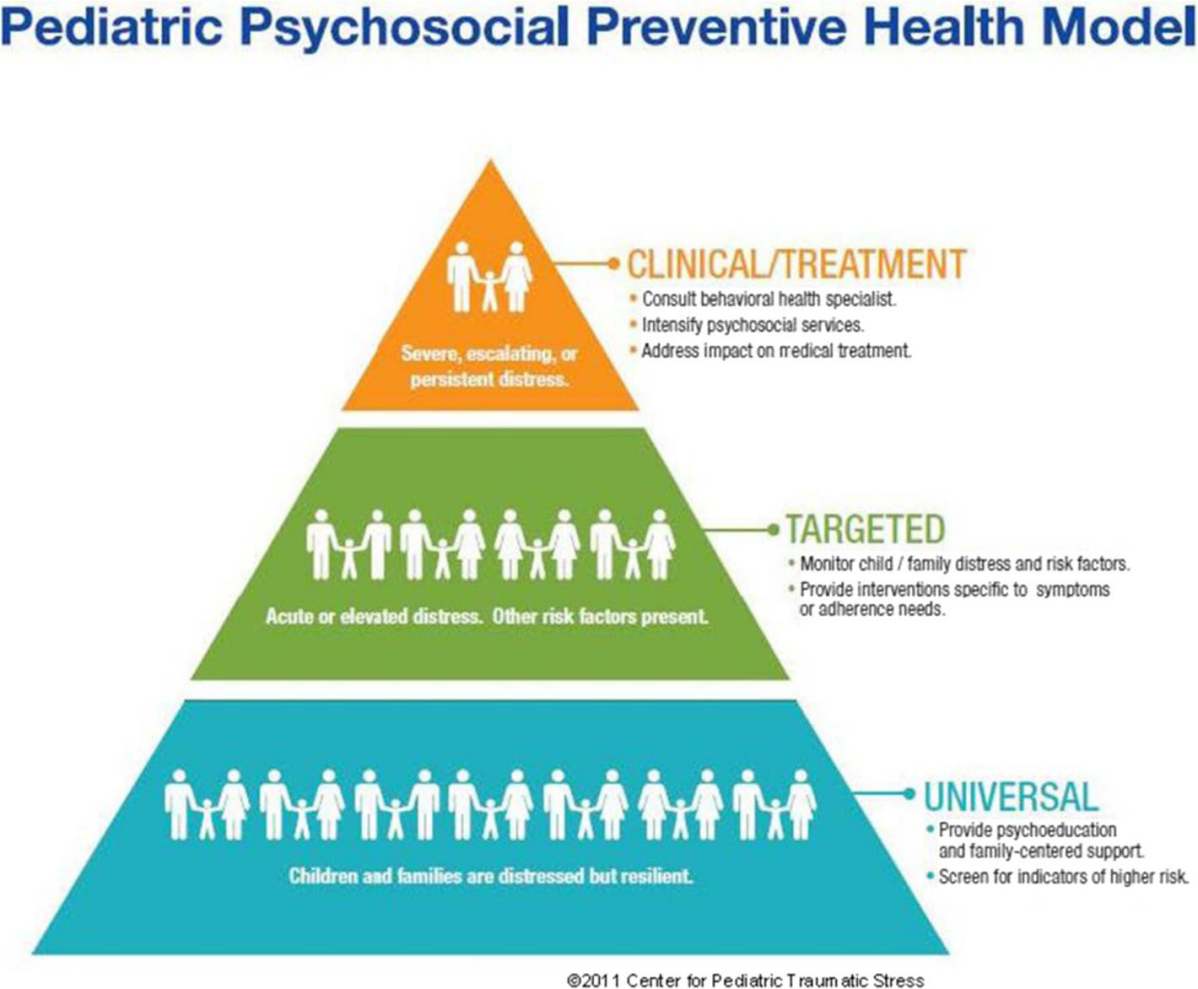
Pediatric Psychosocial Preventative Health Model

The Psychosocial Assessment Tool (PAT) [7, 8] is an evidence-based parent/caregiver report screener of family psychosocial risk in English and Spanish. The PAT generates a total score and 7 subscales (family structure, social support, child problems, sibling problems, family problems, stress reactions, family beliefs). Since the initial versions of the PAT [14–16], we have refined the PAT with the current all literacy version reflecting the broad assessment of family psychosocial risks. Screening with the PAT can be completed at diagnosis [17], can be used by multidisciplinary staff, and facilitates the delivery of psychosocial care [18]. Embedded in social ecology theory, the PAT total score maps on to the Psychosocial Preventative Health Model (PPPHM, Fig. 1) [19], a three-tier model which represents the distribution of psychosocial risks across the population of families. Most families experience some distress but have minimal risk factors (low levels of distress, few prior child, or family problems) and resources (financial resources, strong social support) that help them cope and adapt to their child’s illness (universal). A smaller group of families (targeted) have identified areas of risk and moderate resources. At the top of the pyramid are families with more severe problems, many risk factors, and few resources (clinical).

Empirical evidence supports the readiness of the PAT for broad implementation. It is used in 28.9% of pediatric cancer programs in the USA [11] and widely in other countries [20–28]. The PAT is acceptable to families across race, ethnicity, and SES [7, 8, 10, 16, 17, 29] and has been shown to impact psychosocial outcomes at higher levels of distress when results are shared with staff [30]. We adapted the PAT for hematopoietic stem cell transplantation (HCST) [31] including development of a clinical pathway to facilitate its integration in clinical care [32, 33], and in sickle cell disease, adding items to capture relevant aspects of the social context for families (e.g., school absences, changes in housing) [34].

We do not know the extent to which the PAT has been adopted, whether implementation is consistent across families, and if PAT implementation is sustained. The best practices for implementation have not been studied, and barriers and facilitators to systematic implementation have not been evaluated. Challenges such as time, determining who will screen, technical difficulties, and linking screening to care were potential barriers. In a pilot of an implementation model using a workshop and consultation calls in three states, 9 of 12 centers successfully implemented the web-based PAT, half using both the English and Spanish versions [35]. The pilot data informs the implementation and measurement strategies in this study.

### Specific aims

**Fig. 2:**
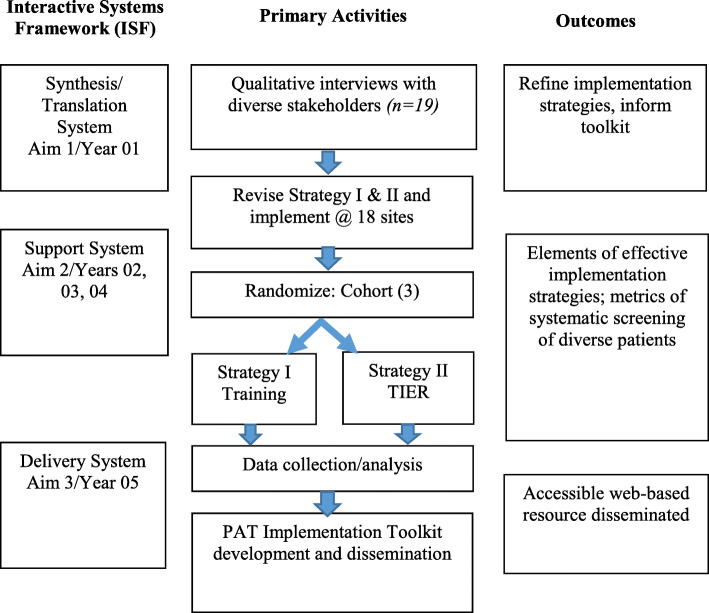
Interactive systems framework and stages of the implementation study

Based on the Interactive Systems Framework for Dissemination and Implementation (ISF) [36], there are three stages in this mixed methods research (Fig. 2). First, two implementation strategies [37], to improve integration of the PAT into standard pediatric cancer care, will be refined using feedback from 19 stakeholders (qualitative methods). The strategies are based on prior PAT studies, the dissemination and implementation literature [37], and Social Ecological [38] and Pediatric Psychosocial Preventative Health Models [19]. Strategy I is training (webinar) to educate providers on the PAT and its administration. Strategy II is Training + Implementation Expanded Resources (TIER), which augments training with consultation calls and identification of a site champion. Second, we will conduct a comparative effectiveness trial of the two strategies at 18 childhood cancer centers of three sizes examining family (penetration, health equity), provider (feasibility, acceptability, burnout, and job satisfaction), and institution (adoption, sustainability, costs) implementation outcomes [39]. We will randomize sites to time of implementation (three cohorts) and strategy (two—I, II). Third, we will develop and disseminate a web-based PAT Implementation Toolkit for family psychosocial risk screening in pediatric cancer.

#### Aim 1

Refine strategies I (training) and II (TIER) using semi-structured interviews with stakeholders—parent advocates, multidisciplinary health care providers, national pediatric oncology professional organizations, and health care industry leaders (implementation team).

#### Aim 2

Compare the two theoretically based and empirically informed strategies to implement the PAT in English and Spanish using a cluster-randomized controlled trial. Compared to training:

**H2.1**. At the patient/family level, TIER will be associated with: (a) a higher proportion of families of newly diagnosed children screened and provided with feedback (penetration) and (b) higher rates of screening for ethnic minority and socioeconomically diverse families (health equity).

**H2.2**. At the provider level, TIER will be (a) more feasible and rated as appropriate and acceptable, (b) associated with greater engagement in addressing health disparities, and (c) associated with less burnout and better job satisfaction.

**H2.3**. At the institution level, TIER will be associated with: (a) a higher rate of site participation (adoption), (b) more positive perceptions of implementation benefits and fewer challenges (sustainability), and (c) psychosocial care better matched to need demonstrating a more equitable distribution of services and costs of care.

#### Aim 3

Based on the results of the trial and further guidance from the implementation team, we will integrate acceptable, feasible, and effective strategies to develop and disseminate a web-based PAT Implementation Toolkit.

## Methods and design

### Overview of the study

The aim of this mixed methods research is to implement universal, systematic family psychosocial risk screening with the PAT in English and Spanish to assure that *all* families of children newly diagnosed with cancer at the participating cancer centers are screened. The setting/context of the research is pediatric cancer programs in the USA. The approach, reflected in the three aims, is guided by the ISF [36]. We selected specific implementation strategies from the Expert Recommendations for Implementing Change (ERIC) project [37] targeting implementation outcomes at three levels (patient/family, provider, institution). ERIC strategies utilized are noted in parentheses throughout this article.

Aim 1 corresponds to the first component of the ISF, Prevention Synthesis and Translation System. We will prepare programs to implement the PAT by conducting qualitative semi-structured interviews with a diverse set of stakeholders (*n* = 19). Interviews will be focused on details of strategy I (training via webinar) and strategy II (training + TIER—consultation calls and identification of a champion), followed by questions about facilitators and barriers, and about implementation strategies and resources needed for universal screening and care delivery to address health inequities. We anticipate fine-tuning and adding components to improve penetration and health equity targets, acceptability and feasibility, adoption, and sustainability.

**Fig. 3:**
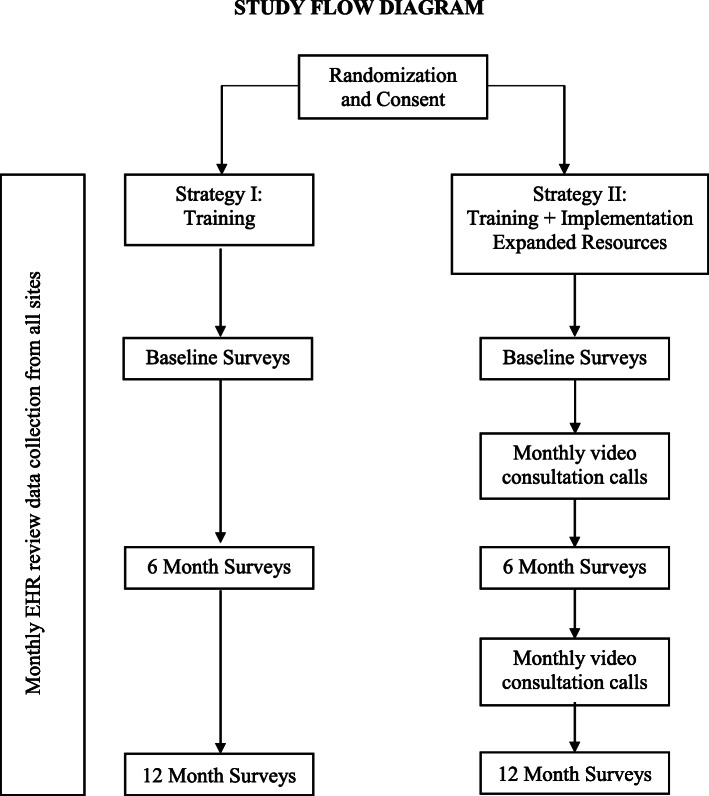
Study flow diagram

The activities of aim 2 correspond to the second component of the ISF, Prevention Support System. Support for those implementing innovation occurs at multiple levels within the system—*patients/families, providers,* and *institution.* To implement the PAT in English and Spanish, 18 pediatric cancer programs, of varying sizes and with geographic distribution assuring representation of ethnic and racial minority families and families at socioeconomic risk, have agreed to participate. We will conduct a cluster-randomized comparative effectiveness trial of the two implementation strategies across three cohorts stratified by size of site based on new patients per year (Fig. 3).

Aim 3 activities correspond to the third component of the ISF, Prevention Delivery System, in the development and dissemination of the PAT Implementation Toolkit.

### Stakeholder interviews to refine implementation strategies (aim 1)

Qualitative interviews will be conducted with a national group of diverse stakeholders, selected using purposive criterion-based sampling [40] to represent different levels of the social ecology. The data from the interviews will inform components of the two implementation strategies and incorporate questions related to broader implementation (e.g., facilitators and barriers). The stakeholder interviews focus on ERIC implementation strategies (use advisory boards and workgroups, prepare patients/consumers to be active participants, and build a coalition).

#### Participants

Participants (*n* = 19) are parent advocates, multidisciplinary healthcare providers, members/leaders of key pediatric oncology professional organizations, and leaders in the pediatric healthcare industry.

#### Procedure

Each stakeholder will be interviewed by video conference using a theoretically driven and empirically based semi-structured interview guide [41]. The purpose of the interview is to refine strategies I and II, identify additional barriers or facilitators of implementation across the patient/family, provider, and institution levels, and ensure PAT implementation strategies address these barriers to reduce disparities in care.

### Cluster-randomized comparative effectiveness trial (aim 2)

PAT implementation will be tested using a head-to-head randomized implementation (comparative effectiveness of two implementation strategies) trial initiated in three cohorts of 1 year each (2021, 2022, 2023). All 18 pediatric cancer programs invited to participate in the trial agreed to a 2-step randomization process stratified by size of site; sites will be randomized to one of three cohorts and then to strategy, comparing strategy I (training) with strategy II (TIER).

#### Selection of sites

The following criteria were used in site selection:

1. *Provide staff and tablet computer for screening*. Each site agreed to provide the staff person(s) screening and tablets. Each site will be provided support for a portion of the site PI’s effort and a part-time research coordinator based on center size for purposes of research only (the research coordinator will not do the screening).
2. *Center size*. Because program size is related to the size of the psychosocial team and related psychosocial resources [5], we used PIPS-CSS data to stratify by size to obtain three equal and clinically relevant groups based on number of new pediatric cancer patients annually—small (30–60), medium (61–149), and large (150+). We selected 6 sites for each of the three size categories.
3. *Psychosocial staff*. To assure that there are staff to conduct screening and act on the results of screening, selected sites are at or above the median for the size of their psychosocial team (number [full time equivalents] social workers + psychologists + psychiatrists + child life specialists) [5] based on PIPS-CSS data.
4. *Diversity of population*. We selected centers in states with a high percentage of ethnic or racial minority families and/or families with socioeconomic disadvantage. CA, TX, FL, NY, and NJ have large Hispanic populations [42]. AL, NC, and VA have the most rapidly increasing Hispanic populations, and AL, LA, NC, and VA have the largest percentage of African-Americans [43]. Thirteen out of 18 sites are in these states. Twelve out of 18 are in states with > 20% of children living in poverty [44]. Other sites were selected to balance geography, race, ethnicity, and SES.

#### Participants

Since screening will be integrated into the clinical services of the centers, it will be routine clinical care. Coded EHR data with minimal protected health information (PHI) will be collected for patients. Reflecting the importance of staff in implementation [45], consented participants include the site PI and screeners and, in strategy II, the champion.

#### Procedure

All sites, regardless of randomization to strategy I or II, will participate in a 3-h professionally prepared webinar (develop educational materials, distribute educational materials) at the beginning of their cohort year. The webinar will include all information necessary to understand, access, and deliver the web-based PAT. The webinar will be based on our in-person training program and curriculum [35] modified to integrate feedback from aim 1 interviews. Each site PI, all screeners, and the research coordinator will participate. For sites randomized to strategy II, the champion will attend. We will work with sites to identify this team and the champion (in TIER) “individuals who dedicate themselves to supporting, marketing, and driving through an implementation, overcoming indifference or resistance that the intervention may encounter in an organization” [37].

After the PAT is completed online, it is scored immediately, and a summary of the score and clinical concerns identified is generated. Only coded data will be transmitted to the study data core. A master list for this data will be maintained at each respective study site and will not be shared with the core research team. We will provide support to sites in the technical aspects of the implementation related to using the web-based forms (centralize technical assistance). If we identify any pattern of problems with technical aspects of implementation, we will communicate with sites promptly.

#### PAT implementation plan

Each site will complete an implementation plan, describing who will be screened, where results will be stored, how results will be communicated to families and to staff, and how results will be used (develop a formal implementation blueprint). For TIER, the plan includes the responsibilities of the champion.

#### Strategy I

For sites randomized to strategy I, the webinar is the implementation condition. Sites will receive technology support, as needed, throughout the implementation period. These strategies correspond to implementation strategies—creating a structure for implementation including creating implementation teams and developing an implementation plan [37].

#### Strategy II

Strategy II includes the webinar and technical support as above with the addition of two evidence-based resources that may improve implementation. The site PI and center staff conducting screening will participate in a monthly 1-h consultation call (provide ongoing consultation, create a learning collaborative) with other TIER sites in that cohort. The group format of this strategy is intended to foster group problem-solving and peer support about issues in implementation. Sites will identify a champion (identify and prepare champions) who will advocate for PAT implementation and support staff in screening activities by serving as a resource to problem-solve and communicate with the broader clinical staff about screening and psychosocial risk. The champion will likely be a clinical leader with enthusiasm and commitment to universal psychosocial risk screening.

#### Measurement/outcomes (all sites)

The measures assess outcomes across patient/family, provider, and institution levels (Table 1) that are clearly operationalized and reproducible [46]. Site PIs and coordinators will attend training on data collection procedures, in separate sessions for the two conditions.

**Table 1:** Measurement

Level/Hyp	Concept	Source	Metric/measure
Patient/family 2.1	Penetration Health equity	EHR (monthly)	Demographics: race, ethnicity, zip code, insurance No. eligible families, English/Spanish No. eligible families screened, English/Spanish % Family feedback letter provided
Provider 2.2	Feasibility Appropriateness Acceptability Engagement in addressing health disparities Job satisfaction Burnout	Survey (Pre, 6-month, Post)	Acceptability of intervention measure Intervention appropriateness measure Feasibility of intervention measure AREA scale of physician engagement Satisfaction employees healthcare Maslach Burnout Inventory version for Medical Personnel
Institution 2.3	Adoption Sustainability: perceived benefit Cost-effectiveness: services/need and cost	Survey	Site participation rate PAT implementation questionnaire
		EHR (monthly)	Psychosocial Services and Medical Treatment Checklist Intensity of Treatment Rating Scale

At the patient/family level (Hyp 2.1) the site coordinator will extract EHR data and send the coded data via REDCap to the data core. The following data for English and Spanish versions of the PAT will be reported monthly: new patients meeting eligibility requirements per the PAT implementation plan; patients with documentation of PAT screening; patients receiving feedback letter; demographic data on all eligible and all screened (race, ethnicity, zip code, insurance).

At the provider level (Hyp 2.2), the site PI and screeners will complete the following self-report measures (~ 15 min) at baseline (T1), and 6 (T2) and 12 (T3) months via REDCap: acceptability of intervention measure; intervention appropriateness measure and feasibility of intervention measure [47]; measure of physician engagement in addressing racial and ethnic health care disparities [48], satisfaction of employees in healthcare survey [49], and Maslach Burnout Inventory [50].

The outcomes at the institution level (Hyp 2.3) are as follows: adoption, the intention of sites to use the PAT (the ratio of sites that initiate the study); whether screening is perceived as an asset to the institution using the PAT Implementation Questionnaire (PIQ) [35] which will be completed by the site PI, the screeners, and the champion at T1, T2, and T3; whether services are matched to the needs of families based on PPPHM levels using EHR data using the Psychosocial Services and Medical Treatment Checklist (PSMTC) [18] at 30, 60, and 90 days; cost using the data from the PSMTC to estimate the amount of psychosocial staff time and national median hourly salary data [51–53]. The Intensity of Treatment Rating Scale (ITR-3) [54] will be completed at the 90-day post-screening data collection time point based on diagnosis, stage, and treatment.

#### Fidelity

Whether the PAT was administered as intended will be assessed using monthly EHR data. The coded data captured at Nemours will also allow us to confirm the completion of each PAT in its entirety. The coordinators will meet monthly to problem-solve any concerns about data collection. In the TIER condition, attendance on calls and minutes of the monthly consultation calls will be prepared. The champion will complete a questionnaire at the beginning of the study year, and at 6 and 12 months to document their activities.

### Dissemination toolkit (aim 3)

To broadly disseminate the PAT, a theoretically and data-driven web-based PAT Implementation Toolkit will be developed with the information, resources, and tools necessary to implement the PAT. This corresponds with the Prevention Delivery System of the ISF (develop educational materials, distribute educational materials, purposely re-examine the implementation) [37].

#### Participants

There will be 20 purposively sampled participants from the aim 1 stakeholders (*n* = 10) and from sites that implemented the PAT in aim 2 (*n* = 10 site PIs and champions). Cognitive interviews will be conducted to ascertain their interpretation and understanding of the PAT Implementation Toolkit. This sample size is consistent with the PROMIS methods [55] and the past experience of the research team [56].

#### Procedure

Subsequent to the final analysis of data from the aim 2 trial, we will refine the preliminary framework for the PAT Implementation Toolkit website which we expect will include the training webinar, technology support, PAT Implementation Plan, complete information to implement the PAT across centers of different sizes, materials specific to using the Spanish version of the PAT, suggestions for overcoming identified barriers and bolstering facilitators, evidence for how screening can impact health disparities, frequently asked questions, and guide for sites in identifying resources to guide intervention based on PAT scores. The Toolkit will be web-based and easily accessible.

#### Cognitive interviews

Qualitative, think-aloud interviews [57] will be conducted by video conference using a semi-structured interview guide. During this audio-recorded interview, providers “walk through” the web-based PAT Implementation Toolkit to identify areas for clarification and improvement. Interviews will be transcribed by a professional transcription service and uploaded to a qualitative data management program (Atlas.ti ©) for formal coding.

#### Dissemination

The Agency for Healthcare Research and Quality Publishing and Communications Guidelines (https://www.ahrq.gov/research/publications/pubcomguide/index.html) will guide dissemination. We will coordinate with Children’s Oncology Group (COG) to assure sites have access to the Toolkit. We will contact all sites in the PIPS-CSS study to support their use of the Toolkit. As a site in the National Cancer Institute Community Oncology Research Program (NCORP), we will include a broad network of community and minority sites.

### Data analysis

#### Aim 1

The interviews will be electronically recorded and professionally transcribed. All data will be stored and managed on a secure drive. Data will be inductively analyzed using Atlas.ti ©. Content analytic strategies will be used to identify codes, categories, and themes [58, 59]. Analysis will proceed as the investigators simultaneously collect information through interviews, read each interview as an individual case, consider the topics addressed in the interview guide, disassemble each interview through coding with preliminary codes, define and combine codes into categories and categories into themes, and consider data from each category across all cases [60].

We will first independently code three interviews selected to represent different stakeholders (patient/family, providers, and institution) to define an initial set of codes and codebook. Study coordinators will be trained to manage and analyze qualitative data [61–63] and supervised by an expert qualitative researcher. The investigators will independently code five interviews. For the remaining interviews, each transcript will be coded independently by a two-person team that will discuss each interview and reconcile any discrepancies until they reach 75% agreement. After this point, interviews will be coded independently but will be reviewed and discussed if a discrepancy is found. The investigators will review all the coded transcripts. As coding continues, codes will be combined into categories. Finally, all data will be examined in each category to combine categories into themes. The themes will be translated into content of the PAT webinar for strategy I and enhanced strategies included in strategy II with attention to health disparities and barriers and facilitators at the patient/family, provider, and institution levels. The rigor of the iterative analytic process will follow standards for qualitative research [63, 64].

#### Aim 2

PAT implementation will be tested using a head-to-head randomized implementation (comparative effectiveness of two implementation strategies) trial in three cohorts over 3 years. *Randomization*. Eighteen sites will be randomized to one of three cohorts. Each cohort will be further randomized to one of the two strategy conditions. Randomization will stratify sites by size to maximize internal validity and statistical power. Sites will be randomized by a data analyst not connected with the study using a two-step Excel = RAND() function. The sequence will be stored in a password-protected electronic file. *Sample size*. The estimated sample of patients/families is based on the median number of new patients each year from the PIPS-CSS database: small (*n* = 60), medium (*n* = 92), large (*n* = 339) multiplied by 6 sites at each size, a total of 2946, with 1473 families allocated to each strategy. With an anticipated Interclass Correlation Coefficient (ICC) [65] of .005, the effective sample size is 950 per arm, a sample size that is sufficient to detect a small effect between the two strategies given 80% power (alpha = .05, one sided). We project a Hispanic sample of 20% of the total (*n* = 589). Based on our research [8], approximately 30% of Hispanic caregivers will be more acculturated/not literate in Spanish at the level necessary to complete the Spanish PAT, rendering a sample of 412. With an anticipated ICC of .005, the effective sample size is 224 which is sufficient to detect a medium effective between the English and Spanish versions given 80% power (alpha = .05, one sided). At the provider level, each of 18 sites has a PI and up to four people screening, and for TIER sites, a champion. Therefore the staff sample ranges from a minimum of 45 ([18 × 2] + 9) to a maximum of 99 ([18 × 5] + 9), likely in the middle given the range of size of sites. At the institution level, 18 sites will be randomly assigned to one of two strategies (9 sites/group). These sample sizes are comparable to other implementation science studies [66].

#### Data cleaning and missing data

All data will be reviewed for valid values/data entry errors, outliers, and extent/pattern of missing data. Descriptive statistics will be reviewed. Consistency and logic checks will be applied for review/cleaning. The multiple group analysis models will provide valid estimates of efficacy if the proportion of missing values is < 10%. Analysis will be conducted at the patient/family, provider, and institution levels. The effect of program size and cohort will be examined and controlled if needed.

- H2.1. ANOVA will compare the effectiveness of the two implementation strategies on penetration and health equity. The outcomes are proportions: families screened/families eligible, families provided feedback/families screened, ethnic minority families screened/ethnic minority families eligible, low SES families screened/low SES families eligible. ICCs among the clusters will be calculated and used to adjust for the cluster effect [67, 68].
- H2.2. Three sets of outcome variables—perception of implementation, engagement in addressing health disparities, and burnout/job satisfaction—will be tested. A two group analysis using Structural Equation Modeling (SEM) will compare the effectiveness of the two strategies [69, 70]. To test the effect of time, we will conduct latent growth curve analysis [71–73]. Analyses will be conducted using Mplus 5.0 [74] with ML estimation for outcome variables that meet the distribution assumptions, and WLSMV estimation for variables that do not. Potential mediating effects of favorable perception of implementation on provider job satisfaction and burnout will be examined using mediation models. TIER is expected to be associated with more favorable perceptions of implementation, less burnout, and higher job satisfaction.
- H2.3. At the institution level, adoption of the PAT will be measured by a ratio of sites that initiate implementation/sites that agreed (H2.3a). If substitutions are necessary, sites that are newly invited will be added to the denominator and adoption calculated by total acceptances/total invited. ANOVA will be conducted to compare the effectiveness of the two strategies. For H2.3b sustainability (PIQ perceptions of implementation benefits and challenges), ANOVA will be conducted to compare the effectiveness of the two strategies on benefits and challenges. For H2.3c, we are interested in the extent to which psychosocial care is matched to need. A cost-effectiveness threshold or criterion to which to compare costs of screening with these two implementation strategies has not been established, necessitating our consideration of valued outcomes in the psychosocial screening literature and resources available. We will use a data analytic approach that we used previously [18]. ANOVA will be conducted to test whether psychosocial care is matched with levels of psychosocial risks, resulting in a 2 (strategy) × 3 (psychosocial risks: clinical, targeted, universal) design on equitable distribution of services and costs of care. It is expected that the 3 PPPHM levels will be related to number and costs of services provided as measured on the PSMTC, with least at universal and most at clinical. It is expected that TIER will result in a better match between level of risk and services provided. Scores from the PSMTC will be derived and mapped onto the levels of the PPPHM. Additional analyses will be conducted to compare English speaking and Spanish speaking families, different ethnicity, race, and SES and insurance status on the outcome variables for H2.3c.

#### Aim 3

We will summarize the data by item and then aggregate the results across participants to reflect potential problems with Toolkit components and to identify components that are clear and supportive of effective implementation [56]. To ensure rigor, we will systematically analyze and then summarize the interview data following a formal coding scheme. Subsequently, we will develop a cognitive interviewing outcome report, a description of the number and type of participants and interviews completed, a description of the specific procedures used in the interviews and the interview guide, and a written summary of feedback on each of the components [56]. This report will be distributed to the study team who will discuss the issues identified. Decisions will be documented in a tracking matrix [75]. Based on this feedback, with aim 2 data and theoretical frameworks, we will revise and finalize the Toolkit.

## Discussion

Family psychosocial screening, a Standard of Care, if implemented consistently and across children’s cancer programs can reduce disparities in care by facilitating care matched to need, and promote adaptation. The aim of this research is to implement universal, systematic family psychosocial risk screening with the PAT in English and Spanish to assure that *all* families of children newly diagnosed with cancer at the participating cancer centers are screened. Guided by the ISF for Dissemination and Implementation [36], this study will result in the development and broad dissemination of a PAT Implementation Toolkit for successful and sustainable implementation of universal, comprehensive, evidence-based family psychosocial screening for all families in pediatric oncology. As one of the few applications of implementation science in pediatric cancer, the results of this trial will provide valuable information about what strategies are effective in supporting comprehensive care. The broad theory proposed by the ISF is innovative in this field and provides a broad, forward looking approach to the process of advancing integrated care.

This project is ambitious and has some potential challenges. If a site that agreed to participate is unavailable, we have other eligible sites at each size to approach. We will retain the balance of sites from states with health disparity populations if we make substitutions. Second, to focus on population-based implementation, we did not consider sites with fewer than 30 new patients annually, and we selected sites with psychosocial staff at the median or above for their size. In addition, our selection criteria (minority population, psychosocial staff) precluded some sections of the USA, particularly less populated states with the smallest centers. Thus, we will not be able to generalize implementation to the smallest sites with fewer resources. However, we will distribute the PAT Implementation Toolkit to these sites and seek to evaluate the impact of broader dissemination of the Toolkit across sites of different sizes and different geographic locations. Finally, for cost-effectiveness, measuring the cost of psychosocial care is complicated and largely without precedent. Involvement of healthcare leaders in aims 1 and 3 will provide expert input in larger system level change to support family psychosocial risk screening and psychosocial care in pediatric cancer.

The importance of evidence-based psychosocial care for children with cancer and their families is recognized. However, too frequently, families of children with cancer do not receive this care, magnifying health disparities in our increasingly diverse pediatric oncology population. These inequities relate directly to screening for family psychosocial risks associated with social and relational determinants of health. Implementation of an evidence-based, parent report screener of family psychosocial risk across the social ecology in English and Spanish may address these health disparities. Risk screening initiates a process of preventive interventions across treatment. Universal, systematic screening assures that assessments are integrated and resources meet the needs of all children with cancer and their families.

## Supplementary information

**Additional file 1.** StaRI checklist

**Additional file 2.** CONSORT checklist

## Data Availability

The datasets used during this study will be made available by the investigators on reasonable request.

## Abbreviations

AIM: Acceptability of Intervention Measure
ANOVA: Analysis of variance
AREA: Awareness, reflection/empowerment, action
COG: Children’s Oncology Group
EHR: Electronic health record
ERIC: Expert Recommendations for Implementing Change
ICC: Interclass correlation coefficient
IRB: Institutional Review Board
ISF: Interactive Systems Framework
ITR: Intensity of Treatment Rating Scale
NCORP: National Cancer Institute Community Oncology Research Program
PAT: Psychosocial Assessment Tool
PHI: Protected health information
PI: Principal investigator
PIPS-CSS: Preparing to Implement the Psychosocial Standards–Current Staffing & Services
PIQ: PAT Implementation Questionnaire
PPPHM: Pediatric Psychosocial Preventative Health Model
PSMTC: Psychosocial Services and Medical Treatment Checklist
REDCap: Research Electronic Data Capture
SEM: Structural equation modeling
SES: Socioeconomic status
StaRI: Standards for Reporting Implementation Studies
TIER: Training + Implementation Expanded Resources
WLSMV: Robust Weighted Least Squares
